# The Effect of Craniosacral Therapy on Blood Levels of Stress Hormones in Male Firefighter Cadets: A Randomized Clinical Trial

**DOI:** 10.3390/bs13110914

**Published:** 2023-11-08

**Authors:** Małgorzata Wójcik, Bruno Bordoni, Idzi Siatkowski, Ewa Żekanowska

**Affiliations:** 1 Department of Physiotherapy, Faculty of Sport Sciences in Gorzow Wielkopolski, Poznan University of Physical Education, 61-871 Poznan, Poland; 2 Department of Cardiology, Foundation Don Carlo Gnocchi IRCCS, Institute of Hospitalization and Care, 20100 Milan, Italy; bordonibruno@hotmail.com; 3 Department of Mathematical and Statistical Methods, Poznan University of Life Science, 60-637 Poznan, Poland; idzi@up.poznan.pl; 4 Department of Pathophysiology, Faculty of Pharmacy, Collegium Medicum in Bydgoszcz, Nicolaus Copernicus University in Torun, 85-094 Bydgoszcz, Poland; zorba@cm.umk.pl

**Keywords:** stress, cortisol, corticotropin-releasing hormone

## Abstract

(1) Background: Fire department cadets preparing to become firefighters and paramedics experience high levels of stress when participating in incidents like traffic accidents and fires. Stress adversely affects health, and coping with it proves difficult. Unfortunately, there is no single method that reduces stress completely in humans. One non-invasive method for lowering stress hormone levels is craniosacral therapy. (2) Methods: Fifty-seven firefighting cadets aged 18–24 years (21.63 ± 1.41) participated in the study. They were randomly assigned to either a test group or a control group. Participants’ blood levels of cortisol and CRH (corticotropin-releasing hormone) were assessed before and after the study. The study group underwent 5-week craniosacral therapy (1× per week). (3) Results: The Kruskal–Wallis test showed that the therapy group’s results were statistically significant for CRH values (*p*-value = 0.00067) and for cortisol values (*p*-value ≤ 0.0001). Wilxocon and Dunn tests showed statistical significance for cortisol after CS therapy between the control and study groups (*p* = 0.0377), and for CRH between the control and study groups before (*p* = 0.00634) and after the study (*p* = 0.000887), and in the study group before and after the study (*p* = 0.0101). (4) Conclusions: The application of craniosacral therapy reduced stress hormone levels in male firefighter cadets. The results indicate that craniosacral therapy (five sessions, one per week) has an effect on the reduction of stress hormones.

## 1. Introduction

Firefighter-rescuers are particularly vulnerable to high levels of anxiety and stress as a result of attending incidents where their health and lives may be at risk. Stress is inherent in the work of a firefighter [1]. Cortisol is an essential mediator between psychological states and health-related outcomes. As the end product of the hypothalamic–pituitary–adrenal (HPA) axis, cortisol helps to regulate the stress response [2]. Perceiving a stressor induces the release of corticotropin-releasing hormone (CRH) from the paraventricular nucleus (PVN) in the hypothalamus [3]. CRH then triggers adrenocorticotropin hormone (ACTH) secretion from the pituitary gland into the bloodstream. ACTH binds to receptors in the adrenal cortex that stimulate the secretion of cortisol [3]. This stress response system optimally regulates stress when a quick onset is required, followed by a swift termination once a threat has passed [2,3]. Short-term psychological stress provides the stimulus for the ‘fight or flight response’ and adaptive hormonal responses to maintain systemic homeostasis, while excessive or chronic stress, through elevated cortisol, can have maladaptive, widespread effects on health [2]. Cortisol has been shown to have an adverse effect on hippocampal and prefrontal cortex neurons, which in turn leads to altered memory and cognitive functioning [4]. Cortisol may also intensify age-related neurodegenerative processes in the brain [4]. Growing evidence indicates that the pathological manifestations of chronic stress include neuronal and synaptic atrophy/malfunction, as well as immunosuppression [4]. Glucocorticoids can have adverse effects on hippocampal and prefrontal neurons, leading to a reduction in the volume of these structures, and dysfunction in them enhances HPA axis activity [5]. In addition, both stress and exogenous glucocorticoids induce behavioral changes that are mainly characteristic of depression and anxiety disorders [6]. In helping a person who is burdened by excessive anxiety, the focus should be on balancing the activity of the autonomic nervous system, reducing muscle tension, and lowering the level of perceived stress [7].

Attention is increasingly being paid to techniques associated with a holistic approach, which means that body and mind should be taken into consideration equally [8]. In Norway, for example, craniosacral therapy has been used as a complementary method for treating people with trauma [8]. What is specific to the cranial area of osteopathy is the extension of osteopathic principles to the cranial sphere, and the synchronization with micro-movements in the tissue and speculative tissue rhythms [9].

Osteopathy in the cranial sphere was developed in the early 1930s by the osteopathic physicians W. G. Sutherland and C. Weaver [9]. The aim of craniosacral therapy is to reduce tension in the cranium, pelvis, diaphragm, thorax, and sacrum, which in turn leads to relaxing the connective tissue structures, in which tension is usually the cause of health problems [8]. Craniosacral therapy is also used to reduce chronic tension-type headaches [10]. Craniosacral therapy is effective for treating pain in patients experiencing neck and back pain, migraine, fibromyalgia, epicondylitis, and pelvic pain [10]. The efficiency of cranial treatments has also been demonstrated in the treatment of premature infants [11]. Another study showed the influence of the CV-4 technique in autonomic-related parameters such as heart rate, blood pressure, blood flow velocity, electroencephalography alpha power, and muscle sympathetic nerve activity [12]. Current evidence demonstrating the effectiveness of craniosacral therapy in reducing stress is promising [13]. It has also been shown that a single Osteopathic Manipulative Treatment (OMT) session of approximately 20 min, with a light touch on the craniosacral body regions, led to a faster recovery of the heart rate and sympathovagal balance in healthy participants, and prevented the typical rise in cortisol levels after a psychological stressor [14].

The aim of our study was to evaluate the effectiveness of craniosacral therapy on levels of serum cortisol and CRH as objective indicators of HPA axis activity.in healthy men (fire service cadets).

Before proceeding with the study, the following research hypothesis was defined:

The groups analyzed, i.e., CS—Craniosacral group and CO—Control group, are statistically significantly different, and the values of Cortisol (C) and CRH (Corticotropin-Releasing Hormone) levels are statistically significantly different between the research group (CS—Craniosacral) and the control group (CO—Control Group) before and after the study.

## 2. Materials and Methods

### 2.1. Participants

Fifty-seven firefighter cadets aged 18–24 (21.63 ± 1.41), with a mean Body Mass Index of 24.44 ± 3.05 kg/m^2^, volunteered to participate in the study. These were only men, as circulating cortisol levels have been shown to be influenced by gender (in particular, the phase of the menstrual cycle in women) [15]. Participants in the study were recruited through meetings held at the fire academy, posters, and leaflets, and they also received comprehensive information about the study project. Those interested in participating in the study were interviewed to check the inclusion and exclusion criteria. Inclusion criteria were male gender and being a fire academy cadet. The exclusion criteria were daily cigarette smoking, alcohol abuse, caffeine use (>300 mg/day), medication, drug use, reported medical illness, and the following types of disorders: endocrine, cardiovascular disease, and psychiatric, physiotherapeutic or osteopathic therapy applied now or in the past, because therapeutic intervention involving, for example, spinal manipulation increases cortisol levels in the body [16,17]. In this study, we were keen to exclude factors that may affect stress hormones. The group of cadets were in a barracks, staying in the same conditions throughout the study period.

### 2.2. Randomization

To assign participants to groups, a block randomization was performed using an Excel file. First, a block was generated to assign sample numbers equally to each group. The block size was randomly generated (2-, 4- or 6-letter combinations of A and B). Following this, each block was assigned to a group. A block randomization allows the distribution of patients in the study groups to be equal in number for the entire study. Depending on the block size adopted (usually 4 or 6), a series of blocks are randomly sequenced that are permutations of possible treatment assignments. Each block should contain an equal number of allocations to each arm (control and experimental). Each participant was provided with a printed number in order that the therapist could not predict which treatment (placebo or craniosacral therapy) would be performed until the participant came.

### 2.3. Cortisol and CRH Assessment

All blood samples were taken at the beginning of the study and at the end of the study, i.e., after five weeks, by a qualified nurse in the doctor’s office at the Fire Cadet School after a 10–12 h overnight rest (including hours of sleep). Blood samples (15 mL) were obtained between 7:00 and 9:00 a.m. from the antecubital vein; Vacutainer tubes were used; the cadets had blood drawn in a sitting position.

Blood was distributed in one tube with gel and a clot activator (10 mL) to obtain serum. Serum was stored at −80 °C until analysis. All laboratory measurements were conducted by medical laboratory scientist. Cortisol levels were measured using an Eagle Biosciences Cortisol ELISA (enzyme-linked immunosorbent assay) kit (cat. DCM020-9) from DiaMetra (Perugia, Italy). CRH levels were measured using an ELISA Kit for Corticotropin-Releasing Hormone (cat. SEC935Hu) from Cloud Clone Corp. (Katy, TX, USA). All the samples from each part of the experiment (pre/post) were analyzed with the same ELISA kit (one kit enables 90 samples to be analyzed); the assay sensitivity was 0.8 nmol/L, and the inter-and intra-assay variation coefficients were all below 10%.

### 2.4. Therapeutic Techniques of Craniosacral Therapy

Before performing craniosacral therapy, all participants in the study group (CS—Craniosacral group) (n = 30) were introduced to the therapy. The therapy sessions took place in a quiet and warm room and were held once a week for five consecutive weeks from 9.30 a.m. to 1 p.m. by the same therapist. Participants lay in a supine position on a couch, and the therapy was conducted each time by the therapist herself, according to a set methodology. In this study, we used a structural approach to craniosacral therapy. The therapist used craniosacral therapy (sacrum compression and traction, AO—Atlanto-occipital joint, mobilization of the frontal bone, parietal bones, sphenoid bone, and temporal bones), and the final step was the CV4 technique—for a full description of the procedure, see [18].

Participants in the no-intervention group (CO—control group) (n = 27) did not receive any therapy. In this group, the therapist only held the subject’s head (while the cadet was in a supinated position) and did not use her hands to apply any techniques; Sutherland’s grip was applied [18]. The therapy time for individual subjects in both groups was 20 min. None of the 57 subjects had received osteopathic or physiotherapeutic therapy prior to the study, and the subjects had no prior knowledge or experience of osteopathic craniosacral treatments.

### 2.5. Statistical Analysis

For the purpose of this research, statistical analysis was performed using R ver. 4.2.2 software [19]. Both the craniosacral therapy experimental group and the control group were analyzed. The values of cortisol and CRH levels in the CS and CO groups are presented as descriptive statistics before and after the research. As the data do not follow a normal distribution, we used the non-parametric Kruskal–Wallis, the Wilxocon test, and the Dunn test with Bonferroni adjustment method. We used the Kruskal–Wallis test to see if the analyzed groups are statistically significantly different, and the Wilxocon test and Dunn test with Bonferroni adjustment to see which groups are statistically significantly different. The results were also presented as boxplots.

## 3. Results

After data collection, statistical analysis was performed using the R package [18]. First, descriptive statistics were determined for the values of cortisol (Table 1) and CRH-corticotropin-releasing hormone (Table 2) in the CS and CO groups before and after the study.

**Figure 1:**
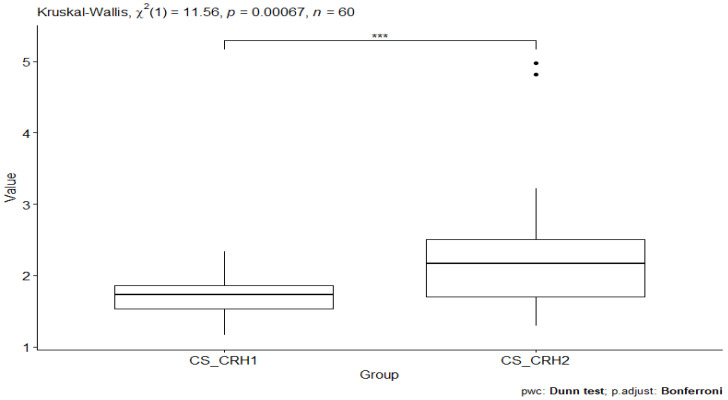
Comparison of CRH values between the CS_CRH1 (CRH value in the craniosacral group before testing) and CS_CRH2 (CRH value in the craniosacral group after testing) groups. Significant codes—***: *p*-value < 0.001.

Further statistical analysis was performed using the Kruskal–Wallis test to check whether the groups being analyzed were statistically different. The result obtained from the analysis of the CS_CRH1 and CS_CRH2 groups indicates the presence of statistical significance, *p*-value = 0.00067 (Figure 1).

**Figure 2:**
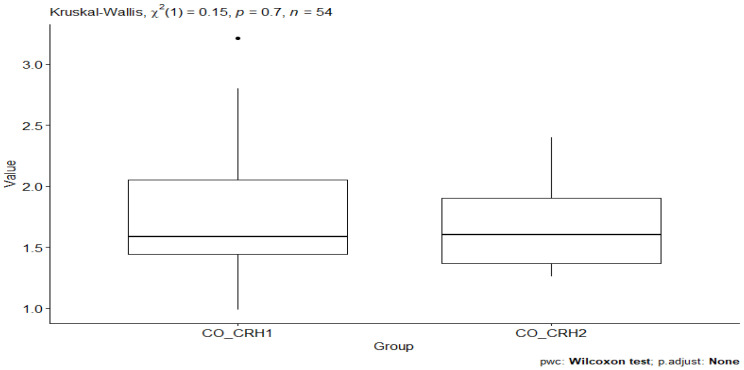
CRH value between CO_CRH1 and CO_CRH_2 groups.

However, comparing the CO_CRH1 (CRH value in the control group before testing) and CO_CRH2 (CRH value in the control group after testing) groups, no statistical significance was observed between the groups’ *p*-value = 0.7. (Figure 2).

**Figure 3:**
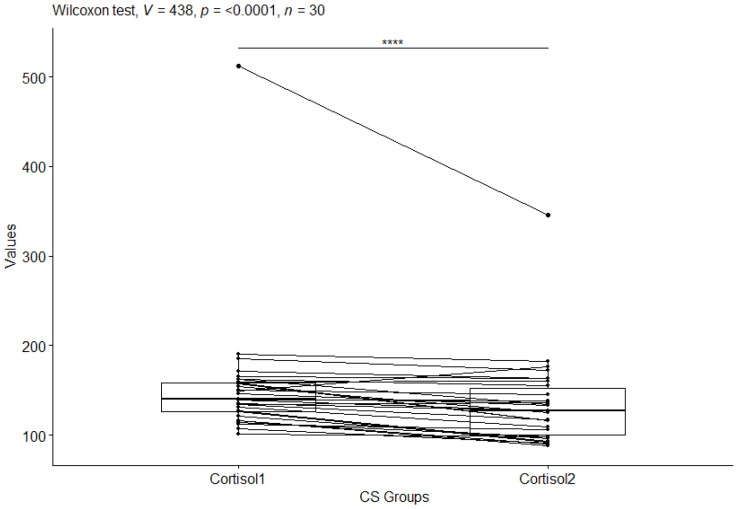
Cortisol values in the craniosacral group before and after the study. Significant codes—****: *p*-value < 0.0001.

**Figure 4:**
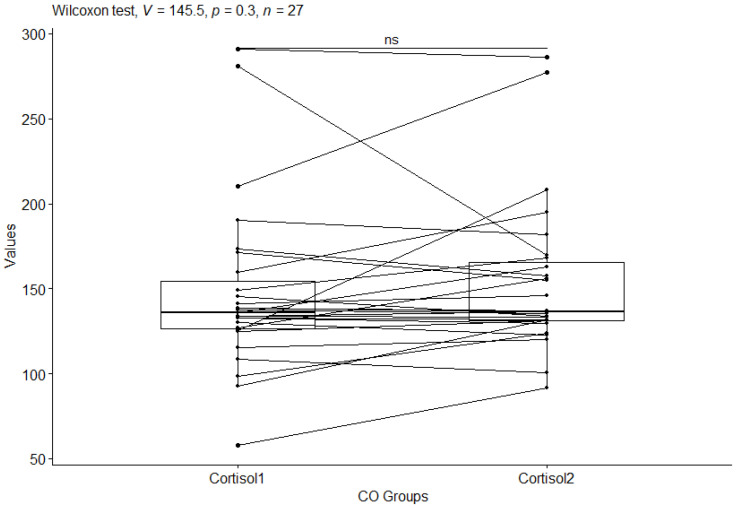
Cortisol values in the control group before and after the study. Significant codes—ns: non significant.

The next step in the statistical analysis was to determine which groups were statistically significantly different, for the CS group before and after the study and for the CO group before and after the study. For this purpose, the Wilxocon test was used. For cortisol (cortisol) values in the craniosacral group, a statistical significance of *p*-value ≤ 0.0001 was observed (Figure 3), while no statistical significance of *p*-value = 0.3 was observed in the control group (Figure 4).

**Figure 5:**
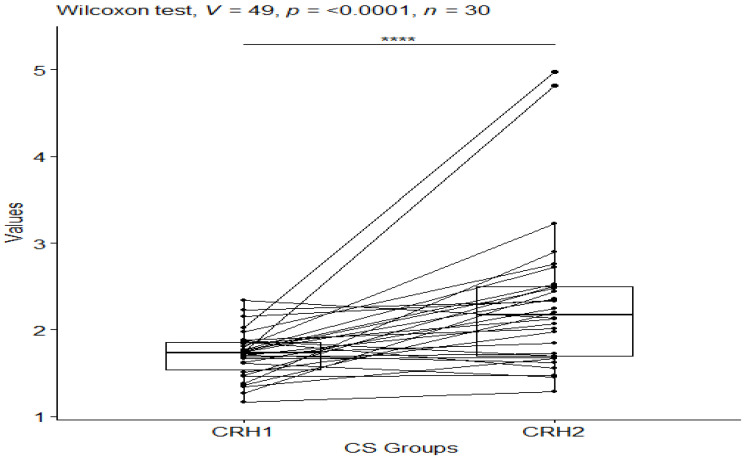
CRH values in the craniosacral group before and after the study. Significant codes—****: *p*-value < 0.0001.

**Figure 6:**
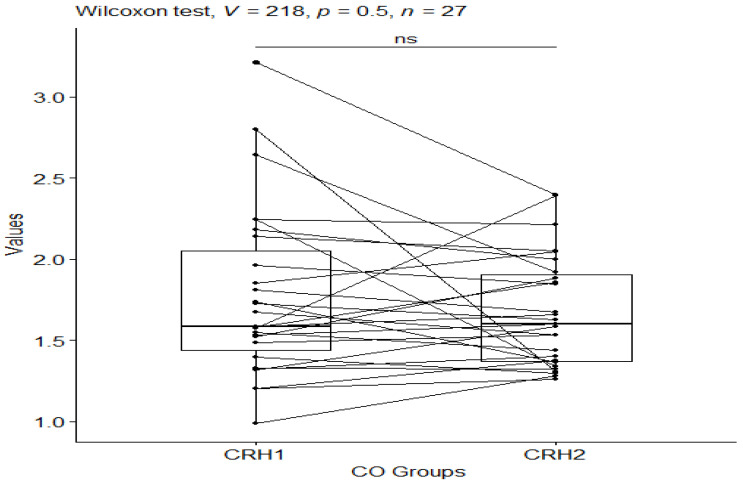
CRH values in the control group before and after the study. Significant codes—ns: non significant.

The Wilcoxon test was also applied to check which groups differed statistically significantly for the CS group before and after testing, and for the CO group before and after testing for CRH (corticotropin-releasing hormone) values. For the CRH values in the CS group, a statistical significance of *p*-value ≤ 0.0001 was observed (Figure 5), while no statistical significance of *p*-value = 0.5 was observed in the control group (Figure 6).

The next step in the statistical analysis was to see which of more than two groups were statistically different for cortisol and CRH (corticotropin-releasing hormone) values. For this purpose, the Dunn test with Bonferroni adjustment was used.

Statistical significance was obtained for cortisol values between the craniosacral and control groups. Statistical significance was obtained for cortisol levels between the CCO2 and CCS2 groups *p* = 0.0377 (Table 3). In contrast, there is no statistical significance for cortisol levels between groups CCO1 and CCO2, CCO1 and CCS1, CCO1 and CCS2, CCO2 and CCS1, and CCS1 and CCS2.

For CRH, statistical significance was obtained between (Table 4) CO_CRH1 and CS_CRH2 *p* = 0.00634; CO_CRH2 and CS_CRH2 *p* = 0.000887; CS_CRH1 and CS_CRH2 *p* = 0.0101. In contrast, there is no statistical significance between (Table 4) groups CO_CRH1 and CO_CRH2, CO_CRH1 and CS_CRH1, CO_CRH2 and CS_CRH1.

## 4. Discussion

The current study was designed to evaluate whether craniosacral therapy might be helpful in reducing stress in male firefighter cadets. Our study demonstrated that craniosacral therapy significantly affected the serum cortisol level and CRH of participants in therapy. Although manual therapies/manual techniques are offered chiefly for chronic tension and the musculoskeletal system, some studies have recommended the use of this therapeutic modality for increasing patients’ general well-being [20]. A prospective study by Edwards et al. showed that osteopathic therapy can indeed be an effective intervention in reducing perceived stress, as well as improving mental health outcomes [21]. Fornari et al. also showed that osteopathic manipulative therapy can improve the effects of conventional therapy for stress [13]. This treatment modality can also induce a relaxation response. For example, Girsberger et al. demonstrated the effect of craniosacral techniques on reducing autonomic nervous system tension [22]. A single session of OMT is able to influence the reduction of autonomic system tension in healthy men [23]. Maintaining a balance between the sympathetic and parasympathetic nervous system is a prerequisite for health [24].

Systemic cortisol is among the most widely used biomarkers of acute and chronic stress [25]. Cortisol contributes to maintaining glucose homeostasis and the cardiovascular system. It is released in a non-specific manner during stressful events. The amygdala, which plays a role in processing the severity of stress, sends a response to the hypothalamus if the level of threat deems it necessary [26]. The hypothalamus then activates the sympathetic nervous system responsible for the fight or flight response. HPA axis overactivity is also a risk factor for cancer [27]. Chronic stress can negatively affect cognitive function [28]. It was observed that, in people with post-traumatic stress disorder (PTSD), yoga had an effect on lowering cortisol, except that, in these studies, cortisol was determined from saliva [28]. Our study showed that craniosacral therapy has an effect on lowering cortisol levels in healthy male subjects, but, in our study, blood cortisol levels were determined. In addition to yoga, aerobic and endurance exercise may also be a way to reduce symptoms of PTSD, as indicated in a review by Reis et al. [29]. Van der Zwan and co-authors showed that exercise and meditation have an effect on lowering stress levels. In this study, stress reduction was tested by assessing heart rate variability (HRV) [30]. Our study also showed the effect of craniosacral therapy on HRV; in addition, we also noted the effect of only grasp-touch on HRV values [31]. Music therapy, too, is proving to be an effective method for lowering stress levels [32] or for use in relaxation methods [33].

The main function of CRH is to stimulate pituitary synthesis [34]. In addition to being produced in the hypothalamus, CRH is also synthesized in peripheral tissues—in particular, the immune system—acting in opposition to cortisol, namely, stimulating the immunological response [35]. CRH is the origin and main driver of the HPA axis; this guarantees that the body has the appropriate stress responses and maintains a heightened state of alertness [7]. Our results show that craniosacral therapy can affect the reduction of CRH levels. The CRH-HPA system affects health, and a disrupted system can cause a condition such as depression [36]. In people with depression or schizophrenia, the continuous effect of CRH on the HPA axis causes increased cortisol, ACTH, and decreased feedback, which results in pathological reactions in the body [37]. The CRH system is associated with serotonergic mediation, emotional imbalance, behavioral changes, anxiety, and depression [36]. For CRH-HPA axis disorders, pharmacotherapy is a common treatment [38]. A study by Zhang et al. used vibratory abdominal massage in insomniacs to show that the treatment affects the HPA axis, affecting CRH production levels and improving sleep [39]. Zhu et al.’s study on mice showed that electroacupuncture affects the reduction in CRH levels [40].

The chronic stress associated with it induces pro-inflammatory changes that are directly linked to the hypothalamic–pituitary axis (HPA), thereby increasing the risk of excessive systemic inflammation [41]. Stress also has an impact on the sympathomedullo–adrenal (SMA) axis. This pathway involves the release of cytokines such as C-reactive protein (CRP), an acute-phase protein released from the liver that increases its response following interleukin-6 (IL-6) secretion. IL-6 is an important pro-inflammatory cytokine. CRP is a sensitive marker in systemic inflammation, and chronically elevated values are an independent risk factor for cardiovascular disease in both children and adults [42].

The firefighting profession is fraught with the incidence of diseases like hypertension and heart attack [43]. Cardiac death is the most common cause of duty-related death among firefighters [44]. Our own research has shown the positive effect of craniosacral therapy on heart rhythm parameters, indicating a reduction in the excitability of the autonomic system [31]. Firefighters are usually exposed to traumatic events while performing their professional duties, putting them at risk of developing post-traumatic stress disorder and depression [43,45].

The incidence of cardiovascular disease and its disability-related effects carries a high financial cost [46]. Prevention appears to be the cheapest form of treatment. Therefore, it seems reasonable to look for non-invasive methods to influence the reduction of stress hormones. Our research shows that craniosacral therapy can be such a method.

Cardiovascular disease cost the European Union (EU) economy EUR 282 billion in 2021 [47]. The financial burden of cardiovascular disease is significant. In Poland, the total costs (direct and indirect) of cardiovascular disease for 2015–2017 range from PLN 34.9 bn (EUR 8.2 bn) to over PLN 40.9 bn (EUR 9.6 bn). Total direct cost and indirect costs were approximately PLN 6.1 bn (EUR 1.4 bn) (16%) and PLN 31.3 bn (EUR 7.3 bn) (84%), respectively [48]. In the USA, median total medical costs for heart failure care were estimated at USD 24,383 per patient [49]. The study by Research Triangle Institute (RTI) International for the American Heart Association predicts that, by 2035 in the USA, cardiovascular disease costs will exceed USD 1 trillion [50]. In Latin America, the cost of heart disease tops USD 30 bn [51].

A very good prevention measure of cardiovascular disease is physical activity, of which firefighters, by virtue of their profession, should have a high level [52]. Physical activity is a well-recognized measure that reduces overweight and obesity and thus counteracts the occurrence of cardiovascular disease [53]. Prevention of cardiovascular disease mainly relies on regular physical activity [54]. Prevention of cardiovascular disease refers to lifestyle changes, including diet [55,56].

Firefighters are also exposed to heat stress, which is associated with cerebral oxygenation and vascular hemodynamics and, in turn, can affect cognitive decline [57]. In the firefighting profession, the development of physical fatigue, which can limit the performance of professional activities, also plays an important role [58]. Stress-induced fatigue can affect neuronal activation patterns, altering them and slowing down simple mental operations [59].

It is very important to prepare an algorithm for general monitoring and coaching systems for firefighters to improve their resilience to stress and reduce risks in their professional activities [60]. The firefighting profession is fraught with the following: high level of stress [61], physical risk, chemical risk, mechanical hazard, and psychosocial risk [62].

The profession of firefighter is carried out by young, healthy, and physically fit people. However, the profession is subject to high levels of stress. It has been proven that people who have experienced traumatic events undergo changes in the hypothalamic–pituitary–adrenal (HPA) axis [63], and firefighters are at risk of post-traumatic stress disorder (PTSD) [64]. The profession is also at risk of experiencing burnout [65]. Research by Vaulerin et al. found that neuroticism in firefighters is associated with three dimensions of burnout [66,67]. Rosca et al. showed that these Dark Triad traits predicted risk-taking at work in firefighters, while altruism, honesty, and courage mediated the relationship between Machiavellianism and risk-taking. Honesty and courage mediated the relationship between psychopathy and risk-taking at work [68].

### Limitations

The relatively small size, convenience, and homogeneity of the sample limit the generalizations that can be made from this study. The intervention was delivered by one practitioner upon healthy young men, which might enhance the observed effect of craniosacral therapy. Moreover, we did not consider female participants, so the results can refer only to men. When considering female participants, such variables as menstrual cycle phase, using contraception and pregnancy/lactation period should be taken into account.

## 5. Conclusions

The results indicate that craniosacral therapy (five sessions, one per week) has an effect on the reduction of stress hormones cortisol and CRH in healthy male firefighter cadets from the studied sample. This method may be an effective and non-invasive way to reduce stress in firefighting cadets. This seems very important, since the firefighting profession is fraught with high levels of stress and the occurrence of diseases as a consequence. Craniosacral therapy appears to be a low-cost and non-invasive way to reduce stress and thus improve quality of life.

## Figures and Tables

**Table 1:** Descriptive statistics of cortisol values in the control group and the craniosacral group: C—cortisol; CO1—control group before the study, CO2—control group after the study; CS1—craniosacral group before the study, CS2—craniosacral group after the study.

Group	Variable	n	Min	Max	Median	iqr	Mean	sd	se	ci
CO1	C	27	58	291	136	28.2	147	49.8	9.59	19.7
CO2	C	27	91.4	286	136	34.2	154	45	8.66	17.8
CS1	C	30	101	512	140	32.4	154	71.3	13	26.6
CS2	C	30	88.2	345	127	52.4	134	48.8	8.91	18.2

**Table 2:** Descriptive statistics of corticotropin-releasing hormone values in the control group and craniosacral group: CRH—Corticotropin-Releasing Hormone; CO_CRH1—Pre-test control group, CO_CRH2—Post-test control group; CS_CRH1—Craniosacral group before test, CS_CRH2—Craniosacral group after test.

Group	Variable	n	Min	Max	Median	iqr	Mean	sd	se	ci
CO_CRH1	CRH	27	0.988	3.21	1.58	0.61	1.77	0.521	0.1	0.206
CO_CRH2	CRH	27	1.26	2.40	1.60	0.533	1.67	0.345	0.066	0.136
CS_CRH1	CRH	30	1.17	2.34	1.73	0.322	1.72	0.274	0.05	0.102
CS_CRH2	CRH	30	1.29	4.98	2.17	0.801	2.31	0.844	0.154	0.315

**Table 3:** Comparison of cortisol values between the craniosacral group and the control group before and after the study (C—cortisol; CCO1—cortisol level in the control group before the study; CCO2—cortisol level in the control group after the study; CCS1—cortisol level in the craniosacral group before the study; CCS2—cortisol level after the study). Significant codes—ns: non significant; *: *p*-value < 0.05.

Group 1	Group 2	n1	n2	Statistic	*p p*.adj	*p*.adj.signif
CCO1	CCO2	27	27	0.675	0.500	0.500 ns
CCO1	CCS1	27	30	0.432	0.665	0.665 ns
CCO1	CCS2	27	30	−1.38	0.166	0.166 ns
CCO2	CCS1	27	30	−0.260	0.795	0.795 ns
CCO2	CCS2	27	30	−2.08	0.0377	0.0377 *
CCS1	CCS2	30	30	−1.87	0.0619	0.0619 ns

**Table 4:** Comparison of corticotropin-releasing hormone values between the craniosacral group and the control group before and after the study (CRH—corticotropin-releasing hormone; CO_CRH1—CRH level in the control group before the study; CO_CRH2—CRH level in the control group after the study; CS_CRH1—CRH level in the craniosacral group before the study; CS_CRH2—CRH level after the study). Significant codes—ns: non significant; ***: *p*-value < 0.001; **: *p*-value < 0.01; *: *p*-value < 0.05.

Group 1	Group 2	n1	n2	Statistic	*p p*.adj	*p*.adj.signif
CO_CRH1	CO_CRH2	27	27	−0.506	0.613	1 ns
CO_CRH1	CS_CRH1	27	30	0.218	0.827	1 ns
CO_CRH1	CS_CRH2	27	30	3.27	0.00106	0.00634 **
CO_CRH2	CS_CRH1	27	30	0.738	0.461	1 ns
CO_CRH2	CS_CRH2	27	30	3.79	0.000148	0.000887 ***
CS_CRH1	CS_CRH2	30	30	3.14	0.00169	0.0101 *

## Data Availability

Data is available from the corresponding author.
